# Structural, optical, and bioimaging characterization of carbon quantum dots solvothermally synthesized from *o*-phenylenediamine

**DOI:** 10.3762/bjnano.14.17

**Published:** 2023-01-30

**Authors:** Zoran M Marković, Milica D Budimir, Martin Danko, Dušan D Milivojević, Pavel Kubat, Danica Z Zmejkoski, Vladimir B Pavlović, Marija M Mojsin, Milena J Stevanović, Biljana M Todorović Marković

**Affiliations:** 1 Institute of Nuclear Sciences „Vinča“-National Institute of the Republic of Serbia, P.O.B. 522, 11001 Belgrade, Serbia; 2 Polymer Institute, Slovak Academy of Sciences, Dubravska cesta 9, 84 541 Bratislava, Slovakia; 3 J. Heyrovsky Institute of Physical Chemistry, Academy of Sciences of the Czech Republic, Dolejškova 3, 182 23, Praha 8, Czech Republic; 4 Faculty of Agriculture, University of Belgrade, Nemanjina 6, 11080 Belgrade-Zemun, Serbia; 5 Institute of Molecular Genetics and Genetic Engineering, University of Belgrade, Vojvode Stepe 444a, 11042 Belgrade 152, Serbia; 6 University of Belgrade, Faculty of Biology, Studentski trg 16, 11000 Belgrade, Serbia; 7 Serbian Academy of Sciences and Arts, Knez Mihailova 35, 11000 Belgrade, Serbia

**Keywords:** antibacterial, bioimaging, carbon quantum dots, precursor, reactive oxygen species

## Abstract

Carbon quantum dots as a novel type of carbon nanomaterials have attracted the attention of many researchers because of their unique optical, antibacterial, and anticancer properties as well as their biocompatibility. In this study, for the first time, carbon quantum dots were prepared from *o*-phenylenediamine dissolved in toluene by a solvothermal route. Subsequently, the prepared carbon quantum dots were encapsulated into polyurethane films by a swelling–encapsulation–shrink method. Analyses of the results obtained by different characterization methods (AFM, TEM, EDS, FTIR, photoluminescence, and EPR) indicate the significant influence of the precursor on structural, chemical, and optical properties. Antibacterial and cytotoxicity tests showed that these dots did not have any antibacterial potential, because of the low extent of reactive oxygen species production, and showed low dark cytotoxicity. By investigating the cellular uptake, it was established that these dots penetrated the HeLa cells and could be used as probes for bioimaging.

## Introduction

Carbon quantum dots (CQDs) as a novel class of carbon nanomaterials can be prepared by using different methods and precursors [1–2]. Most of the common preparation procedures are bottom-up methods [3–4]. Depending on the used precursors and solvents, the structure of the CQDs can be modified significantly, especially, by the presence and distribution of various functional groups on the basal plane and edges of carbon network, affecting, in turn, the CQD properties. Doping of CQDs with nitrogen, chlorine, or fluorine heteroatoms induces larger a transport bandgap, increased charge transfer resistance, and better antioxidant properties compared to pristine CQDs [5]. Functionalization of CQDs with amino groups (NH_2_ groups) induces a redshift of the photoluminescence because of the charge transfer from the amino groups to the carbon honeycomb core [6]. Also, grafting with NH_2_ groups, by means of amines such as 2-ethylenediamine, poly(ethyleneamine), or trimethylamine, enhances the affinity of CQDs to biological structures whereas incorporation of nitrogen atoms in the honeycomb structure of CQDs contributes to a reduction of photobleaching [7]. Tepliakov et al. reported that CQDs can be treated as polymer-like nanoparticles of amorphous carbon with embedded partially sp^2^-hybridized atomic domains [8]. In this structure, electrons are partially delocalized over the domain area, but a strong coupling of the amorphous host matrix is maintained continuously.

CQDs are well known as chemically and thermally stable, quasi-spherical, photoluminescent material with very good antibacterial and anticancer properties under visible light irradiation [9–16]. This material has very good biocompatibility, including low dark cytotoxicity and good cell proliferation. Photoluminescence of CQDs can be tuned, and quantum dots emit light in the range from blue to red. Some of them have very good prooxidant and antioxidant properties [14]. Under blue light irradiation, CQDs produce reactive oxygen species (ROS), which cause oxidative stress and further bacterial death [17–21]. Because of the low dark cytotoxicity they can be used successfully for bioimaging of different cells [22–23]. In our previous research, we investigated the effect of various parameters on structural, optical, and biomedical properties of CQDs [5]. We reported how heteroatom dopants (nitrogen, chlorine, and fluorine) affected structural properties and ROS production with or without visible light irradiation. In addition, we examined antibacterial and cytotoxic properties. An important issue is the preparation of CQDs and polymer-based composites and their possible antibacterial activity including the usage in wound healing [24]. Different authors prepared CQDs by using various precursors and reported on their excellent antibacterial activity and good biocompatibility [25–29]. In this study *o*-phenylenediamine dissolved in toluene was used as precursor for CQDs synthesis by a solvothermal method. The prepared CQDs were encapsulated into medical grade polyurethane (PU) films by a swelling–encapsulation–shrink method. It was investigated how the precursor influenced the structure (morphology and chemical composition) and further prooxidative, antibacterial, and cytotoxic properties of CQDs and CQD/polyurethane composites.

## Results and Discussion

### Surface morphology

**Figure 1:**
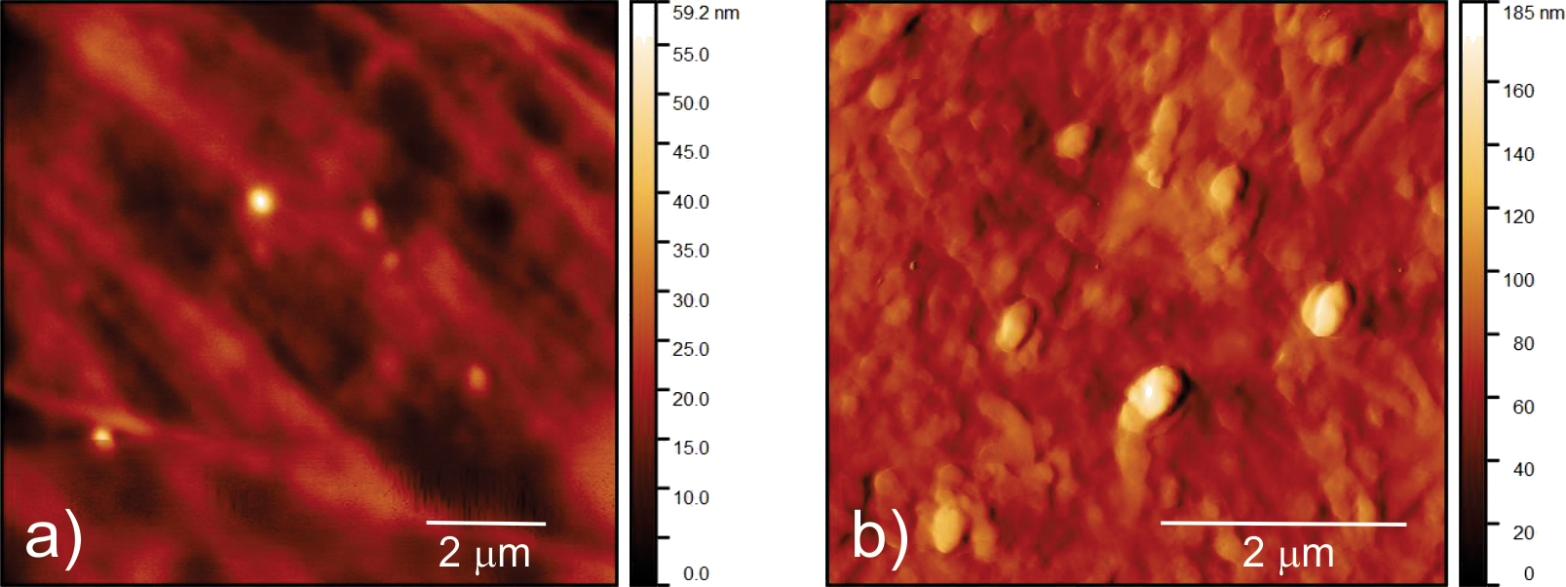
(a) Top-view AFM image of neat PU and (b) top-view AFM image of a CQDs/PU composite.

Figure S1 (Supporting Information File 1) presents the surface morphology of the CQD samples. Figure S1a shows a TEM micrograph of CQDs. The average diameter of these dots is 4 ± 1 nm. A top-view AFM image of CQDs is presented in Figure S1b (Supporting Information File 1). TEM and AFM images show that the CQDs are spherical. Statistical analysis conducted on more than 20 AFM images in Gwyddion software showed that more than 80% of the CQDs had a diameter between 2 and 5 nm while their height was 2.6 nm (Figure S1c,d in Supporting Information File 1). Figure 1 shows the surface morphology of neat PU and a CQDs/PU sample. The RMS roughness values of these samples are 4.45 and 14.04 nm, respectively.

### FTIR, UV–vis, and PL spectra of CQDs and CQDs/PU

To study the chemical and optical properties of CQDs, FTIR, UV–vis, and PL spectra were measured. A FTIR spectrum of the CQDs is presented in Figure S2a (Supporting Information File 1). The spectrum contains many peaks associated with the following bonds: Peaks at 3634 and 3448 cm^−1^ stem from O–H stretching vibrations. A peak at 3367 cm^−1^ could be assigned to N–H stretching vibrations of aliphatic primary amines whereas the peaks at 2880 and 2943 cm^−1^ originate from C–H stretching vibrations. A peak at 1759 cm^−1^ stems from C=O stretching vibrations (carboxylic acid) whereas the peaks at 1696 and 1593 cm^−1^ could be assigned to C=N stretching and N–H bending vibrations, respectively. The peaks at 1521 and 1061 cm^−1^ stem from N–O stretching and C–O stretching vibrations, respectively. The peaks at 961, 815, and 759 cm^−1^ could be assigned to C=C bending, C-H bending, and C=C bending vibrations, respectively [30]. An et al. reported that XPS analysis of CQDs prepared from *o*-phenylenediamine showed the presence of sp^2^ domains predominantly in the carbon core structure of the CQDs, which contributed to the formation of polyaniline fluorophores [31]. Figure S2b (Supporting Information File 1) shows a UV–vis spectrum of CQDs. We can observe that the toluene solution of CQDs has a strong broad absorption band at 248 nm with a shoulder at 224 nm, which represents the π–π* transition of C=C bonds. Apart from this broad band, there is a shoulder peak at 330 nm corresponding to the n–π* transition of C=O [32]. In the visible light region, at 444 nm, there is a weak absorption peak caused by surface state of CQDs [33–34]. Figure S2c (Supporting Information File 1) shows PL spectra of CQDs. It is obvious that the emission of CQDs does not depend on the excitation wavelength. Regardless of the excitation wavelengths the emission is located at 545 nm. Thus, CQDs prepared from *o*-phenylenediamine emit green light and their photoluminescence is excitation-independent. The highest PL intensity can be observed with an excitation wavelength of 490 nm.

**Figure 2:**
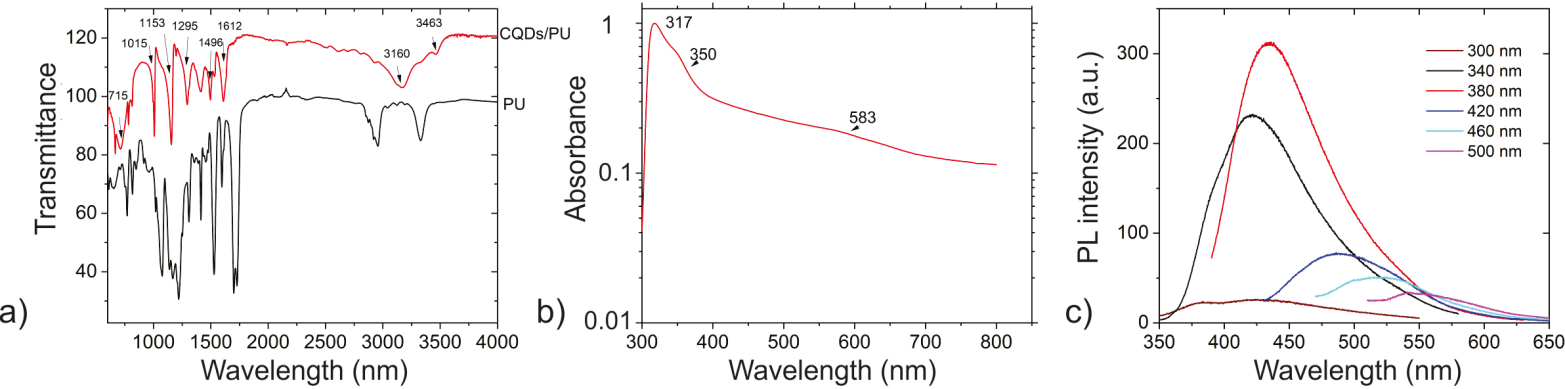
(a) FTIR spectra of PU and CQDs/PU; (b) UV–vis spectrum of CQDs/PU; (c) PL spectra of CQDs/PU.

The PL of CQDs can be tuned by modifying different factors affecting the structure of CQDs, namely surface states, precursors, preparation methods, and heteroatom doping [9]. Previous investigations showed that possible mechanisms of the CQD photoluminescence are radiative recombination of electron–hole pairs in the sp^2^ domains inside the sp^3^ matrix as well as the effect of zig-zag edges [6,18]. Apart from this, surface defects can cause a redshift of the PL emission [35]. Based on the recorded PL spectra, we can conclude that the PL of these dots is dominantly governed by the core states in the conjugated π domains and the quantum confinement effect. Similar to other semiconducting quantum dots (QDs) of nanometer scale, the CQD edges influence the electronic structure of the conjugated sp^2^ domains [35–36]. Figure 2 shows FTIR, UV–vis and PL spectra of CQDs/PU composite samples. It is obvious from Figure 2a that there are some additional peaks in the CQDs/PU FTIR spectrum compared to that of neat PU. The peaks at 3463 and 3160 cm^−1^ belong to O–H stretching vibrations whereas a peak at 1612 cm^−1^ stems from C=C stretching vibrations. A peak at 1496 cm^−1^ could be assigned to N–O stretching vibrations whereas the peaks at 1295 and 1153 cm^−1^ belong to C–O stretching vibrations. The peaks at 715 and 1015 cm^−1^ stem from C=C bending vibrations [30]. All other peaks identified in both neat PU and CQDs/PU composite samples originate from PU.

Figure 2b shows a UV–vis spectrum of the CQDs/PU composite sample. The first peak at 317 nm is due to the π–π* transition of C=C. A peak at 350 nm is due to n–π* transition of C=O whereas a very wide band at 585 nm stems from surface states of the CQDs. All of these peaks can be identified also in the UV–vis spectrum of pure CQDs as well, but when the CQDs are encapsulated inside the polymer matrix, these peaks are redshifted because of the crosslinking of CQDs with polymer chains. Figure 2c shows the PL intensity of the CQDs/PU composite sample. The PL intensity depends on the excitation wavelength while the PL intensity of neat CQDs showed wavelength-independent behavior. After encapsulation of the CQDs into a polymer matrix, PL emission spectra of CQDs are blueshifted compared to the PL emission of neat CQDs, and the highest PL emission intensity is measured at 430 nm for an excitation wavelength of 380 nm (CQDs/PU samples emit blue light). In this way, the PL of CQDs/PU samples is affected by the quantum confinement effect.

### Reactive oxygen species production

#### Singlet oxygen generation

**Figure 3:**
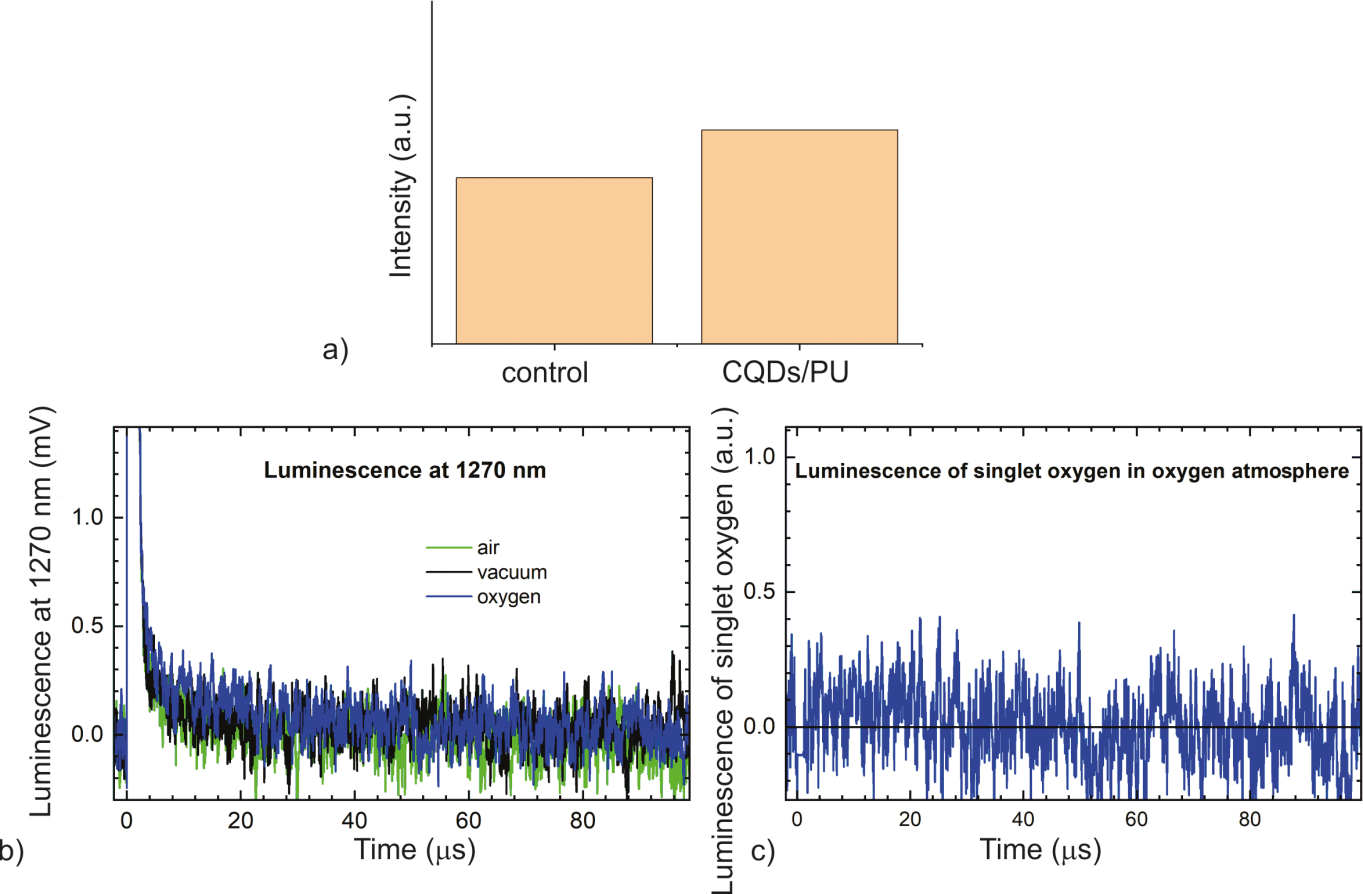
(a) Intensity of the EPR signal of control (left) and CQDs/PU composite sample (right); (b) luminescence of the CQDs/PU composite at 1270 nm in different atmospheres; (c) singlet oxygen luminescence of the CQDs/PU composite in oxygen atmosphere calculated as the difference between traces in oxygen atmosphere and vacuum.

The ability to produce reactive oxygen species (ROS) is a very important parameter for the determination of antibacterial activity of certain material. First, we examined the ROS generation of CQDs (Figure S2d, Supporting Information File 1). From this figure it is obvious that CQDs do not generate singlet oxygen. Further, we examined the ROS production of neat PU (control) and CQDs/PU composite samples by three methods, namely EPR, luminescence at 1270 nm, and UV–vis probe measurement. Figure 3a presents the intensity of EPR signals of control and CQDs/PU composite samples. The figure shows that the CQDs/PU composite samples do not generate singlet oxygen. Second, luminescence measurements were carried out at 1270 nm in different atmospheres, namely air, vacuum, and oxygen (Figure 3b,c). The results show that there is no singlet oxygen generation in any atmosphere. The singlet oxygen production of the CQDs/PU composite sample was additionally investigated through an established photochemical procedure based on the use of 1,3-diphenylisobenzofuran (DPBF) as an efficient quencher of ^1^O_2_ (Figure S3, Supporting Information File 1). This figure shows that the absorption of DPBF solution in both vials is nearly identical. This means that the CQDs/PU sample does not produce singlet oxygen, which confirms the previous results obtained by EPR and luminescence at 1270 nm. Ge et al. reported earlier that graphene quantum dots generate singlet oxygen through energy transfer to molecular oxygen [21]. Chong et al. claimed that superoxide anions are involved in the generation of singlet oxygen, implying that electron transfer is an intermediate step for the generation of singlet oxygen by photoexcited graphene quantum dots [20]. In nitrogen-doped graphene, depending on the doping procedure, the nitrogen moieties include graphitic N together with pyrrolic and pyridinic nitrogen and amino groups [37–39]. Bianco et al. reported recently that pyridine nitrogen can be a reactive center and activates other reactive centers at the adjacent carbon atoms in functionalized C–N bonds for additional post reactions such as oxidations [40]. Obtained FTIR and EDS results indicate that in the CQDs synthesized from *o*-phenylenediamine, NH_2_ groups are dominantly bonded to the basal plane and the edges of the CQDs whereas pyrrolic and pyridinic nitrogen play only a minor role. Furthermore, during the hydrothermal synthesis of CQDs from *o*-phenylenediamine, the used precursor was able to form slowly a thermodynamically stable polyaniline and further conjugated sp^2^ domains with NH_2_ groups. Thus, the formed CQDs do not have reactive centers to generate singlet oxygen or any other reactive oxygen species. In our previous works, we used polyoxyethylene−polyoxypropylene−polyoxyethylene Pluronic 68 (PF68) as precursor to synthesize hydrophobic CQDs [18–19]. These quantum dots produce high levels of singlet oxygen, and their chemical composition differs from that of dots synthesized from *o*-phenylenediamine. They contain aromatic rings bonded with oxygen functional groups, which are distributed over the basal plane and edges, but they do not have any NH_2_ groups or pyrrolic and pyridinic nitrogen. According to Ge et al., CQDs prepared from polyoxyethylene−polyoxypropylene−polyoxyethylene Pluronic 68 generate singlet oxygen through energy transfer to molecular oxygen [21]. But CQDs prepared from *o*-phenylenediamine do not generate singlet oxygen or OH radicals through energy or electron transfer, because the condensation process of these dots includes NH_2_ groups in their structure whereas the presence of pyrrolic and pyridinic nitrogen is really minor. Thus reaction centers for ROS generation (dominantly pyridinic N) do not exist in *o*-phenylenediamine CQDs [40].

#### Hydroxyl radical production

**Figure 4:**
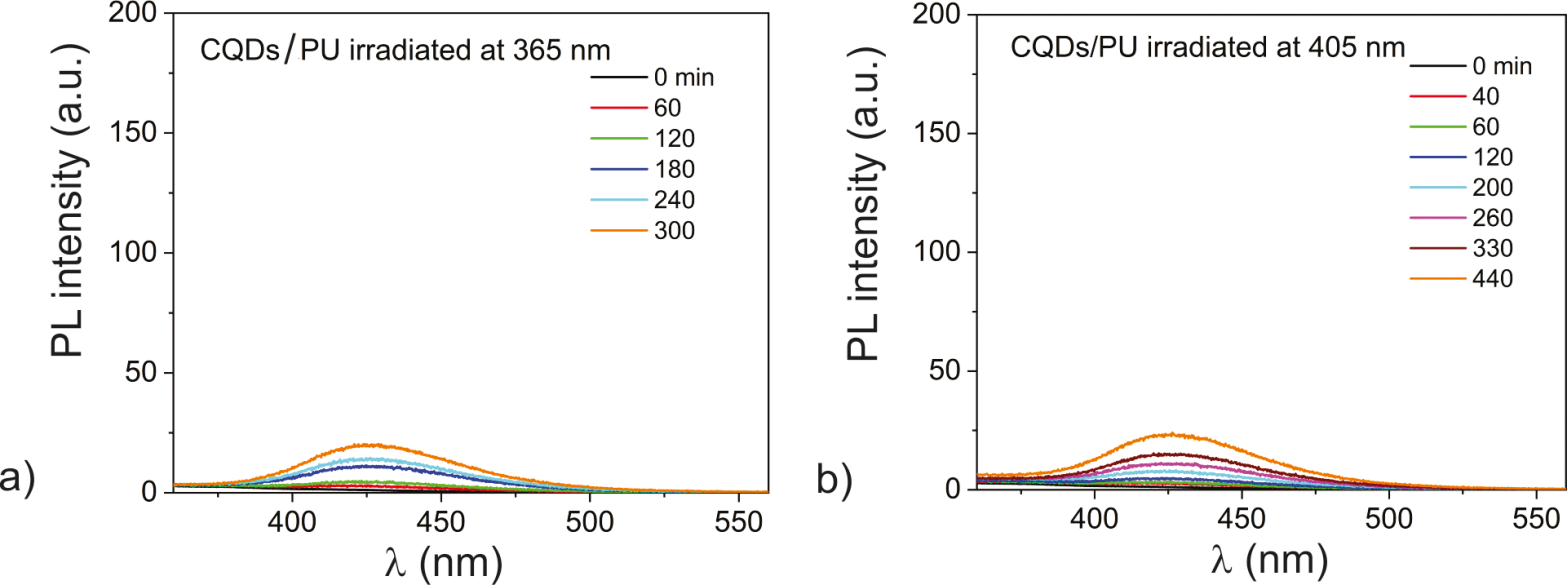
(a) PL spectra of h-TA at different times under 365 nm excitation with 6 mW/cm^2^ intensity; (b) PL spectra of h-TA at different times under 405 nm excitation with 40 mW/cm^2^ intensity.

To examine the production of hydroxyl radicals, two measurements at excitation wavelengths of 365 and 405 nm were conducted. In Figure 4, PL spectra of hydroxyterephthalic acid (h-TA) at different times under 365 nm excitation with 6 mW/cm^2^ intensity and PL spectra of h-TA at different times under 405 nm excitation with 40 mW/cm^2^ intensity are presented.

The obtained results indicate a low level of PL intensity after all measured time periods (0‒300 min) and (0‒440 min). This fact shows that there is no production of hydroxyl radicals in the CQDs/PU composite samples at the two excitation wavelengths (365 and 405 nm). In the previous section we established that the CQDs do not generate singlet oxygen. Both obtained results related to production of ROS indicate that there are no reactive centers in the structure of the carbon core that could enable the production of ROS.

### Antibacterial testing

The antibacterial activity of CQDs differs from the antibacterial mechanism of commercially available antibiotics. The main parameters that determine the antibacterial action of CQDs are generation of reactive oxygen species, cytoplasm leakage due to DNA binding, and gene expression modulation [28]. In addition, the CQD surface charge affects very much the antibacterial activity of CQDs [41]. Recently, Bing et al. observed that CQDs with different surface charges had different antibacterial activities. Positively charged CQDs damaged the membrane of *E. coli* completely whereas negatively charged CQDs interacted only weakly with the bacterial membrane [42]. Uncharged CQDs did not show any antibacterial activity against *E. coli* and *B. suptilis*. In this study, antibacterial testing of all samples was conducted against two bacterial strains, namely *S. aureus* and *E. coli*. The results presented in Table S1 (Supporting Information File 1) showed that CQDs/PU composites prepared from *o*-phenylenediamine did not exhibit any antibacterial activity against *E. coli* or *S. aureus* even after treatment under blue light for 360 min.

These results agree with the results presented in the sections above. The CQDs did not generate any type of ROS. They are uncharged as well. The presence of NH_2_ groups on their surface can possibly contribute to antibacterial activity. NH_2_ groups adsorb onto the bacterial membrane and molecules bearing this functional group can diffuse into the cell interior, where the disruption of the cytoplasmic membrane finally leads to cell death [25,43]. The dots synthesized from *o*-phenylenediamine did not disrupt the cytoplasmic membrane.

### Cytotoxicity testing

**Figure 5:**
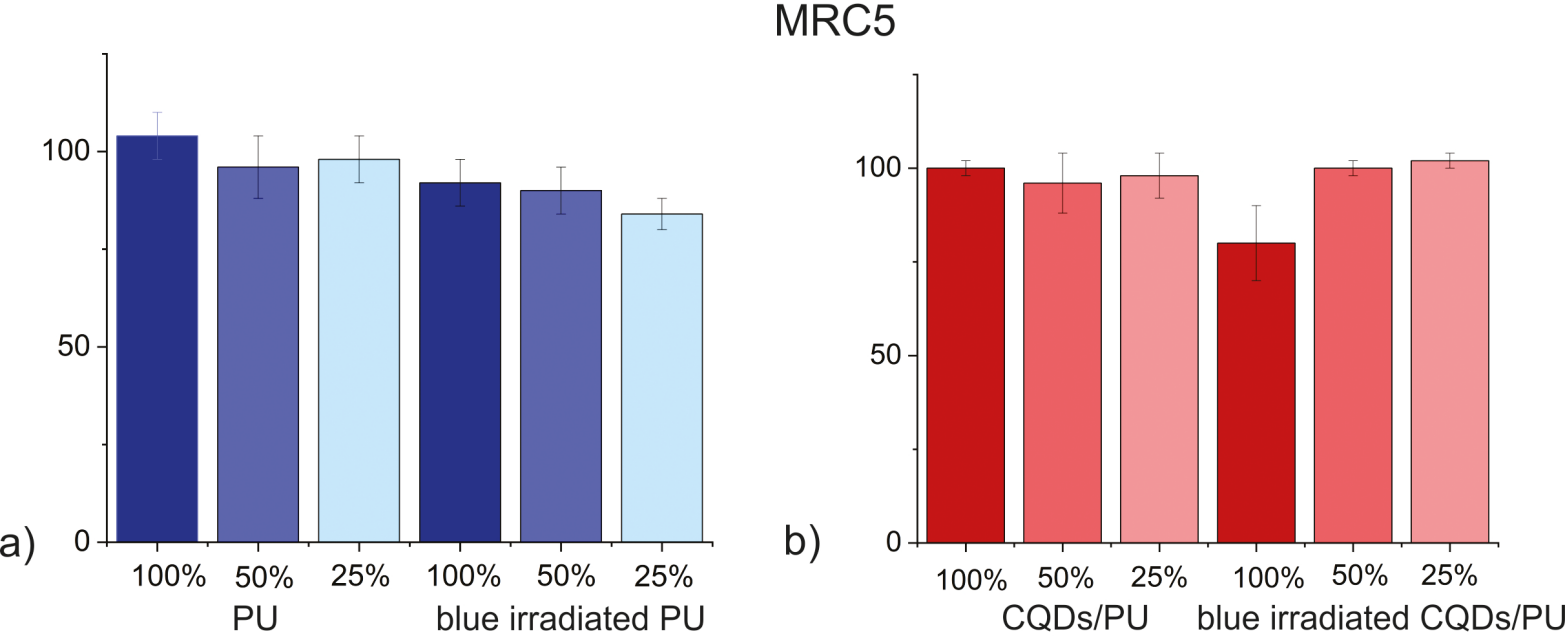
Viability of MRC5 after treatment with PU control (a) and CQDs/PU composite samples (b) using different extract concentrations. No cytotoxicity effect of 50% of extract in the cultivation medium of the CQDs/PU composite on MRC5 cells was detected. All tests were carried out in triplicate.

Low cytotoxicity is one of the mandatory requirements for biomedical applications. In this paper, we performed cell viability tests by applying the MTT assay toward MRC5 human lung fibroblast cells. Lung fibroblasts are very important for maintaining the integrity of the alveolar structure by proliferating and repairing injured areas [44]. MRC5 cells have normal karyotype and are commonly used for genetic, cytotoxicity, viral infection, and other fibroblast-based assays [45]. These cells produce hepatocyte growth factor (HGF), express α-smooth muscle actin, and are used to study the regulation of HGF production and the pathogenesis of tissue fibrosis [46–49]. Figure 5 presents cell viability measurements of individual samples with different extract concentrations. The results are presented as percentage of the control (untreated cells), which was arbitrarily set to 100%. As it can be seen from this figure, none of the tested samples (control and CQDs/PU) showed any cytotoxicity against MRC5 cells regardless of the extract concentration. During the measurements, three different extract concentrations were used, and neat PU control and CQDs/PU composite samples were tested with and without blue irradiation. We established that both MRC5 cells and tested bacteria (*S. aureus* and *E. coli*) exhibited almost equal resistance to CQDs/PU composites. Our previous research showed that CQDs/PU composites had different effectiveness on bacteria and tested cells (adenocarcinomic human alveolar basal epithelial cells-A549 and mouse embryonic fibroblast cell line-NIH/3T3) [18]. By comparing the results of these two investigations, we concluded that the surface chemistry of the encapsulated CQDs in polymer composites has a crucial effect on the properties of CQDs/PU composites.

### Cellular uptake

**Figure 6:**
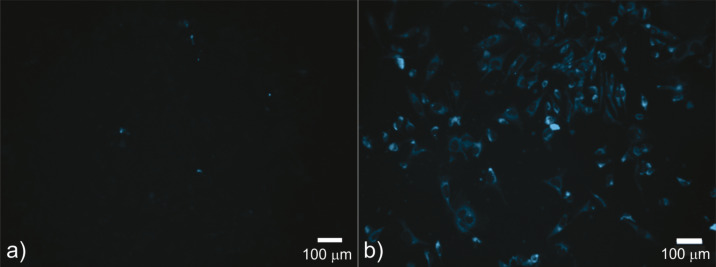
Fluorescence images of (a) HeLa cells treated with vehicle control and (b) HeLa cells treated with CQDs (200 µg/mL) for 48 h. Scale bar is 100 µm.

Photobleaching limits the use of hydrophobic probes (e.g., Nile red) [50]. CQDs can be used as probes for bioimaging because of the tuneable strong photoluminescence and high resistance to photobleaching. In order to test internalization of CQDs, Hela cells were treated for 48 h with a concentration of 200 µg/mL CQDs. As shown in Figure 6b, fluorescence imaging demonstrated that CQDs penetrated Hela cells well (compared to Hela cells treated with vehicle control, shown in Figure 6a) and were mainly in the cytoplasm region (Figure 6b). The obtained results indicate that these dots can be used for visualization of different cell lines because of the low cytotoxicity and poor antibacterial activity.

## Conclusion

In this study we discussed for the first time the effect of the precursor *o*-phenylenediamine on structural, chemical, optical, antibacterial, and cytotoxic properties of CQDs. Nitrogen substitutional doping in the π basal plane of CQDs has been used to modulate their properties. Nitrogen moieties mainly include graphitic N, combined with pyrrolic N, pyridinic N, and amino groups. By using different techniques, we established that N atoms were bonded to the carbon core network dominantly through amino groups. Because of the lack of reaction centers (introduced dominantly via pyridinic N) these dots have very weak potential to generate ROS and also poor antibacterial activity. Due to low dark cytotoxicity these CQDs can be very good candidates for bioimaging.

## Experimental

### Synthesis and characterization of CQDs and CQDs/polyurethane composite samples

CQDs were prepared by a solvothermal method from 0.9 g of *o*-phenylenediamine (Sigma-Aldrich, Germany) dissolved in 50 mL toluene (Sigma-Aldrich, Germany) for 12 h at 180 °C. All chemicals were purchased and used as received. After synthesis, CQDs were filtered through polytetrafluoroethylene (PTFE) filters with 100 nm pore size. The prepared hydrophobic CQDs (toluene solution, 20 mL) were encapsulated into medical grade polyurethane (PU) films (dimension 1 × 1 cm^2^, thickness of 1mm, donated by American Polyfilm) by the swelling–encapsulation–shrink method described previously for 12 h [18]. Later, these CQDs/PU films were dried in a Memmert vacuum furnace at 80 °C overnight to remove toluene. The concentration of the CQDs in the toluene solution was 2.2 mg/mL. These specimens were designated as CQDs/PU. For bioimaging studies, toluene was evaporated, and a thin film of CQDs was redissolved in water and filtered.

The prepared QCD samples were characterized by transmission electron microscopy (TEM), atomic force microscopy (AFM), Fourier-transformed infrared spectroscopy (FTIR), UV–vis spectrophotometry, photoluminescence spectroscopy (PL), and electron paramagnetic resonance (EPR). CQDs/PU composite samples were characterized by UV–vis, AFM, FTIR, PL, EPR, and luminescence measurements.

For TEM imaging (JEOL JEM-1400 operated at 120 kV), CQDs were deposited on graphene oxide copper grids with 300 mesh by drop casting. For AFM imaging, all CQDs samples were deposited on freshly cleaved mica. The AFM measurements were conducted using a Quesant microscope operating in tapping mode in air at ambient temperature. Statistical analysis of all AFM images was performed in Gwyddion software [51]. FTIR measurements were conducted using an infrared microscope Nicolet iN10 Thermofisher Scientific operated in the ATR mode. All samples were deposited on Si substrates by drop casting. Spectra were recorded at ambient temperature in the range of 400 to 4000 cm^−1^ with a spectral resolution of 4 cm^−1^. UV–vis spectra were recorded on a LLG-UNISPEC2 spectrophotometer (LLG, Germany) in the range of 190 to 900 nm at ambient temperature. The PL intensity measurements of CQDs and CQDs/PU composite samples were performed on a RF-5301PC spectrofluorometer (Shimadzu, Japan).

### Reactive oxygen species (ROS) production

#### Singlet oxygen generation

For EPR measurements, a Spectrometer MiniScope 300, Magnettech, Berlin, Germany was used. The instrument was operating at a nominal frequency of 9.5 GHz. 2,2,6,6-tetramethylpiperidine (TEMP) was used as a spin trap. The CQDs/PU samples were dipped in a solution of TEMP in ethanol at a concentration of 30 mM and irradiated with a 3 W blue LED for 12 h.

Time-resolved near-infrared luminescence of O_2_ (^1^Δg) at 1270 nm after excitation by Quantel Q-smart 450 Nd YAG laser (wavelength of 355 nm, pulse length of ca. 5 ns) was observed using a custom-made detector unit (interference filter, Ge diode). The resulting signal was an average of 2000 traces, and it was calculated as the difference between the luminescence of individual samples in oxygen atmosphere and vacuum. The resulting luminescence traces were fitted by a single exponential function excluding the initial part influenced by light scattering, fluorescence, and the formation of singlet oxygen from excited states of CQDs.

Singlet oxygen generation was investigated by UV–vis probe measurements of solutions of 1,3- diphenylisobenzofuran (DPBF) ethanol (20 µM) as well [52–53]. Neat PU and CQDs/PU (1 × 1cm^2^) were dipped in 20 mL of DBPF solution. These solutions were irradiated under blue light (470 nm, 3 W) in a time range of 0 to 30 min, and the absorption of DBPF at 415 nm was recorded using a LLG-UNISPEC2 spectrophotometer (LLG, Germany). The distance between blue light source and the 20 mL vials with samples was 5 cm.

#### Hydroxyl radical production

The CQDs/PU composite samples in the form of 1 × 1 × 0.1 cm^3^ rectangular slabs were immersed in 2 mL of terephthalic acid (TA, Sigma-Aldrich) water/NaOH (2 × 10^−3^ mol/L) solution. The concentration of TA was 5 × 10^−4^ mol/L. Samples were irradiated at 365 and 405 nm with intensities of 6 mW/cm^2^ and 40 mW/cm^2^, respectively.

The fluorescence of the formed hydroxyterephthalic acid (h-TA) solutions taken after different times of irradiation was measured in 1 × 1 cm^2^ quartz cuvettes in a right-angle arrangement. The excitation wavelength was 330 nm.

### Antibacterial testing

International standard ISO 22196 (Plastics – Measurement of antibacterial activity on plastic surfaces) was used to examine the antibacterial activity of CQDs/PU composites [54]. The following strains were used for testing: *Staphylococcus aureus* ATCC 25923 and *Escherichia coli* ATCC 1175. Antibacterial tests were repeated three times while untreated samples were exposed to appropriate controls (three to measure viable cells initially and three to measure viable cells after incubation for 24 h). The sample dimension was 25 × 25 mm^2^ and all of them were washed with ethanol and sterilized by UV irradiation for 30 min. The samples were divided into two groups, that is, one group used as control to check bacterial viability and another group exposed to blue light for different periods of time. 0.2 mL of a bacterial suspension of 6 × 10^5^ cells/mL was used to inoculate the samples. Test inoculums were covered with films (20 × 20 mm^2^), and Petri dishes with test samples were incubated for 24 h at 36 °C. 10 mL of SCDLP was used to recover the bacteria and aliquots of 1 mL with different dilutions were plated on NB agar and incubated for 40 to 48 h. After that time bacterial colonies were counted.

### Cytotoxicity

In order to assess the cytotoxicity of CQDs/PU (antiproliferative activity), standard MTT assay and methods suitable for materials testing were used [55–56]. All tests were carried out in triplicate. Human lung fibroblasts (MRC5) cells (obtained from ATCC culture collection) were plated in 96-well flat-bottom plates at a concentration of 1 × 10^4^ cells/well, grown in a humidified atmosphere of 95% air and 5% CO_2_ at 37 °C, and maintained as monolayer cultures in RPMI-1640 medium supplemented with 100 µg/mL streptomycin, 100 U/mL penicillin, and 10% (v/v) fetal bovine serum (FBS). Material samples were washed with ethanol and UV sterilized for 30 min. For all materials, untreated and treated (60 min under blue light), sample extracts were prepared by incubating samples (1 mg/mL) in RPMI-1640 medium for 72 h at 37 °C. RPMI-1640 medium, in which MRC5 cells were maintained as monolayer cultures, was replaced with undiluted sample extract (100%), and 50% and 25% sample extracts (prepared by diluting sample extracts with fresh RPMI-1640). After 48 h of treatment with sample extracts, cell proliferation was determined using MTT reduction assay by measuring the absorbance at 540 nm on Tecan Infinite 200 Pro multiplate reader (Tecan Group, Männedorf, Switzerland). The cytotoxicity results were presented as a percentage of the control (untreated cells), which was arbitrary set to 100%.

### Fluorescence microscopy

Hela cells were grown in low-glucose Dulbecco's Modified Eagle's Medium (DMEM) supplemented with 10% fetal bovine serum, non-essential amino acids, and penicillin/streptomycin solution (10,000 units penicillin and 10 mg/mL streptomycin) (all from Thermo Fisher Scientific). Cells were maintained in a humidified incubator at 37 °C with 5% CO_2_. 5 × 10^4^ Hela cells were seeded on cover slips in 12-well plates and incubated overnight to allow cells to attach. The next day, cells were treated with medium containing aqueous solution of CQDs at a final concentration of 200 µg/mL (deionized water was used as a negative vehicle control). After 48 h of treatment, the cells were washed in PBS and visualized using an Olympus BX51 fluorescence microscope with a Spectrum Aqua filter. All images were captured using a 20× objective and analyzed with Cytovision 3.1 software (Applied Imaging Corporation).

## Supporting Information

File 1 Additional experimental data.
