# Supplementary material for: Structural, optical, and bioimaging characterization of carbon quantum dots solvothermally synthesized from o-phenylenediamine
Source: Beilstein J Nanotechnol. 2023 Jan 30;14:165–74. doi: 10.3762/bjnano.14.17 (PMC9907016; doi:10.3762/bjnano.14.17)
Supplement: File 1 — Additional experimental data. [file Beilstein_J_Nanotechnol-14-165-s001.pdf]

BEILSTEIN JOURNAL OF NANOTECHNOLOGY

Supporting Information

for

Structural, optical, and bioimaging characterization of carbon quantum dots solvothermally synthesized from *o*-phenylenediamine

Zoran M. Marković, Milica D. Budimir, Martin Danko, Dušan D. Milivojević, Pavel Kubat, Danica Z. Zmejkoski, Vladimir B. Pavlović, Marija M. Mojsin, Milena J. Stevanović and Biljana M. Todorović Marković

Beilstein J. Nanotechnol. **2023,** *14,* 165–174. doi:10.3762/bjnano.14.17

Additional experimental data

License and Terms: This is a supporting information file under the terms of the Creative Commons Attribution License (https://creativecommons.org/licenses/by/4.0). Please note that the reuse, redistribution and reproduction in particular requires that the author(s) and source are credited and that individual graphics may be subject to special legal provisions.
The license is subject to the *Beilstein Journal of Nanotechnology* terms and conditions: (https://www.beilstein-journals.org/bjnano/terms)

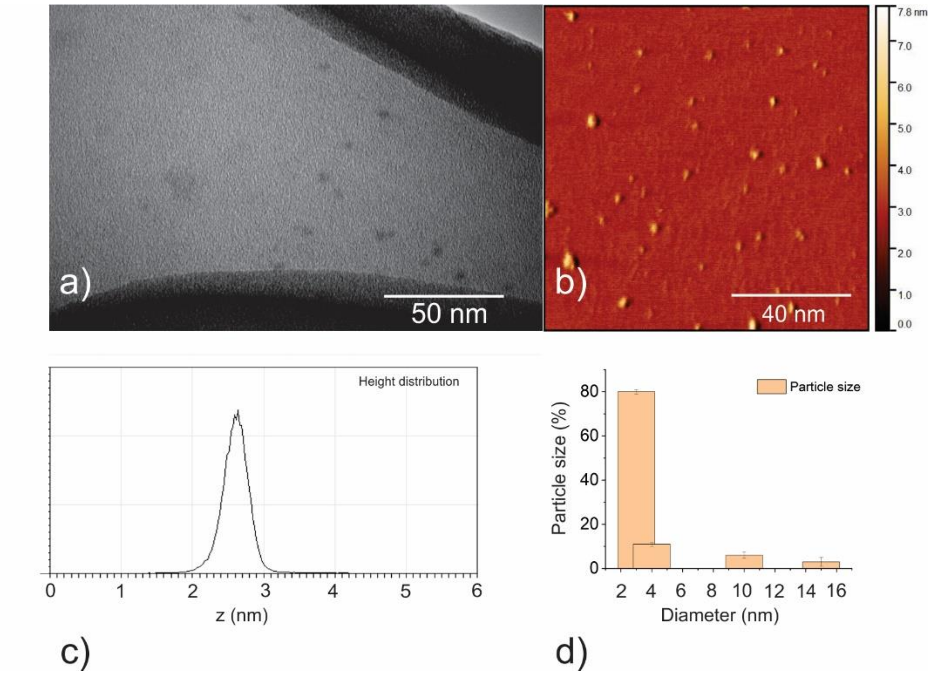

Figure S1: (a) TEM micrograph of CQDs, (b) top view AFM image of CQDs, (c) height profile of CQDs, and (d) particle size distribution of CQDs.

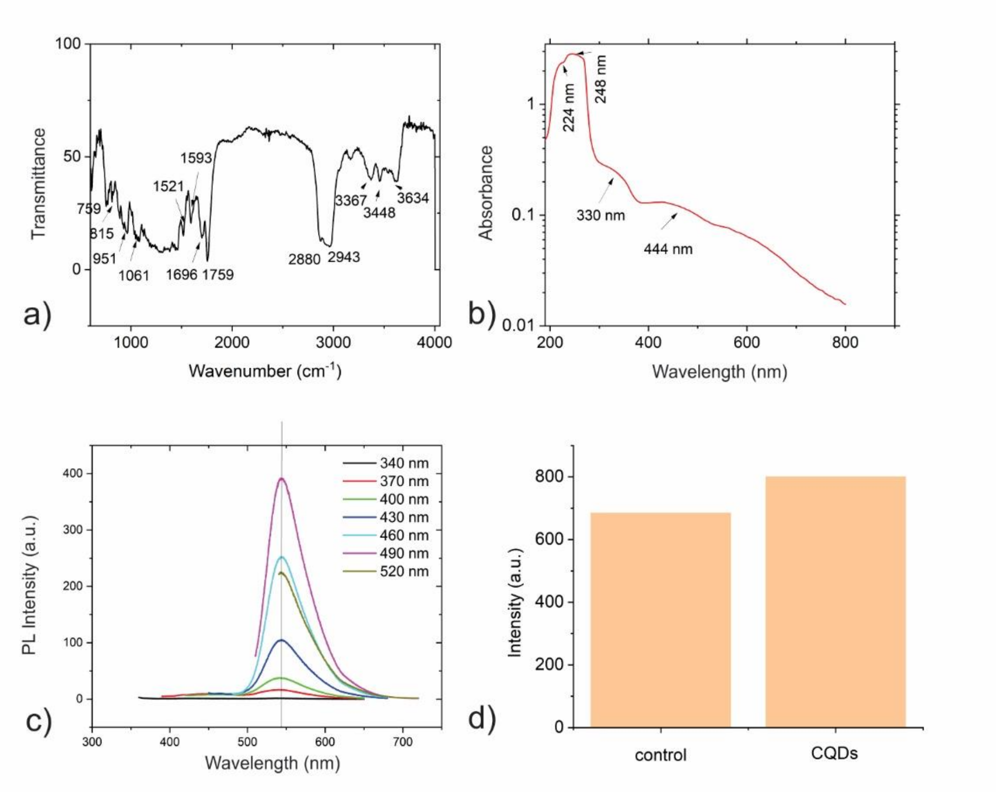

Figure S2: (a) FTIR spectrum of CQDs, (b) UV–vis spectrum of CQDs, (c) PL spectra of CQDs, and (d) EPR spectra of CQDs samples.

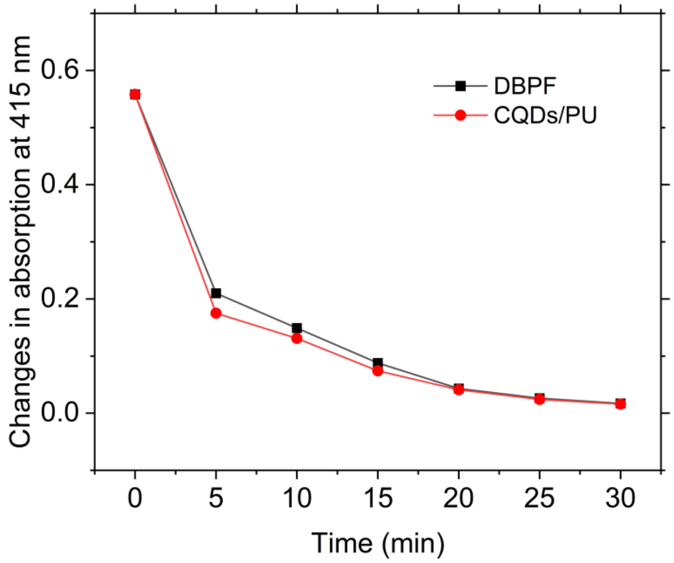

Figure S3: Changes in absorption at 415 nm of DBPF (black curve) and CQDs/PU composite sample (red curve).

Table S1: Antibacterial activity of CQDs/PU composite samples.

Bacteria strains	Irradiation time (min)	Antibacterial activity
E. coli	15	no
	30	no
	60	no
	360	no
S. aureus	60	no
	120	no
	240	no
	360	no
